# Is Impression Management Through Status Updates Successful? Meta-Accuracy and Judgment Accuracy of Big Five Personality Traits Based on Status Updates From Social Network Sites in China

**DOI:** 10.3389/fpsyg.2019.01192

**Published:** 2019-05-24

**Authors:** Ting Wu, Yong Zheng

**Affiliations:** Key Laboratory of Cognition and Personality (Ministry of Education), Southwest University, Chongqing, China

**Keywords:** status updates, personality judgment, impression management, meta-accuracy, judgment accuracy

## Abstract

Status updates on social network sites (SNSs) as a new medium for people to express “what is on your mind” on the Internet can provide much information. In the current study, we statistically analyzed survey data to examine whether individuals utilize impression management in their status updates on SNSs, whether their attempts at impression management are successful, and whether users who post these status updates can infer how others view them based on these contents, whether the status updates posted on SNSs reflect information about users’ Big Five personality traits. The findings suggested that the meta-perception and perceivers’ judgments of all five traits are quite accurate, despite users’ aim to create different impression of most traits in their status updates. This study offers new empirical evidence about the model of interpersonal perception on SNSs and shows that status updates on SNSs can provide considerable information about their authors.

## Introduction

WeChat (*weixin* or 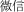 in Chinese) and Tencent QQ (also known as QQ) are two of the most popular social network sites (SNSs) among the younger generation in China (Tencent, 2017). Moments (Circle of Friends or 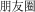 in Chinese) and Zone (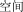 in Chinese) are the main functions of WeChat and Tencent QQ, respectively, both platforms enable users to post status updates.

Status updates are a method for individuals to express “what is on your mind” on the Internet, including the events of their daily life; emotional states, or views on a certain topic, or to share music, movies, or articles that they appreciate. These messages are presented to their online friends and can be “liked” and commented on; thus, status updates are a new form of “one-to-many communication” (Deters and Mehl, 2013) that has become increasingly prevalent worldwide.

Prior research in different national contexts has demonstrated strong associations between personality traits and status updates on one’s Facebook; for instance, narcissism was an important predictor of the frequency of status updates, and perceived self-efficacy was negatively related to the appropriateness of postings (Winter et al., 2014). Relative to introverts, extraverts more frequently update their status, click to share, react to others’ “likes” and comments (Lee et al., 2014), and post updates about their social activities and everyday life more frequently on Facebook; people with higher levels of openness are more likely to post updates about intellectual topics, whereas people with high conscientiousness are more likely to post updates about children, and people who are low in self-esteem are more likely to post updates related to their romantic partners (Marshall et al., 2015). However, prior research has mainly focused on the connection between Facebook and users’ personality. People in Mainland China cannot use Facebook; instead, they use WeChat and QQ. There are some differences between these platforms; for example, in WeChat Moments, if two of your friends are not friends with each other, they can never view each other’s comments on your status updates, and therefore, to some extent, WeChat Moments might be considered more private. QQ Zone has also now added this setting. Given this privacy feature, users might feel free to present themselves more realistically in their status updates. However, these platforms also have many similarities, such as status updates, these platforms allow users to post and enable their friends to comment and “like”; these basic functions are common to the platforms. Although Chinese people cannot use Facebook, they can present themselves via their local SNS platforms—WeChat and QQ. Therefore, we believe that previous research on Facebook can also be extended to a Chinese setting.

Considerable previous research has mainly focused on the connections between status updates and personality traits, particularly on Facebook. In contrast, few researchers have explored impression management in status updates and the meta-perception of posters and personality judgment of perceivers based on this content, especially in the Chinese cultural context. The goal of this paper is to (1) examine whether participants aim to make a certain impression regarding their Big Five personality traits in their status updates and whether these impression management attempts are successful, (2) detect whether participants who post status updates can accurately infer how others judge their Big Five personality traits based on these posts, and (3) investigate whether observers can judge participants’ Big Five personality traits based on the content of their status updates.

### Theoretical Background

According to Gosling et al. (2002) model, the mechanisms linking individuals to the inhabitable environment can be categorized into the following: *identity claims* and *behavioral residue*. Further, Vazire and Gosling (2004) state that this can be extended to the virtual environment. Identity claims are those symbolic statements derived from individuals about how they wish to be perceived; these statements can either be used to convey information to others or be directed at individuals themselves. Behavioral residue refers to the physical traces of a person’s behaviors that are left behind unintentionally (Gosling et al., 2002; Vazire and Gosling, 2004). In a virtual environment, users’ identity claims may be more salient. Owing to the unique affordances of status updates, users may have plenty of opportunities to edit the language of their status updates and carefully choose content to make certain statements about themselves or convey messages to viewers; status updates can thus be regarded as a distinctive type of identity claim in the virtual environment. Exploring the model of interpersonal perception via status updates on SNSs can further enhance our comprehension of the users’ own and others’ perception of the Big Five personality traits through users’ status updates.

#### Impression Management and Self-Presentation in an Online Context

Humans desire to be perceived positively; therefore, they frequently engage in impression management. Impression management is the process by which people control the impressions others form of them (Goffman, 1959; Kowalski and Leary, 1990). Further, many studies also suggest that one of the most important motives for people in an online context is self-expression and impression management (Krämer and Winter, 2008). One popular way for people to manage impressions is by posting photographs on the Internet to display their physical appearance. When users post their photos on a dating website or a professional-networking site, they tend to use relative physical position to manage impressions. For example, women tended to take and display photographs portraying themselves in a relatively low physical position to emphasize youthfulness and attractiveness, whereas men were more likely to take and display photographs portraying themselves in a relatively high physical position to highlight their physical size and dominance (Makhanova et al., 2017). For portraits on LinkedIn (a professional network), gender-related self-expression encourages users to display portraits that highlight their uniqueness and attractiveness (Tifferet and Vilnai–Yavetz, 2018). Pounders et al. (2016) also demonstrated that users who posted selfies were motived to express happiness and physical appearance. As for impression management of personality traits in an online context, a study by Vazire and Gosling (2004) showed that non-acquainted perceivers’ impressions of extraversion and agreeableness were enhanced based on users’ personal websites. Stopfer et al. (2014) also revealed that unique impression management indeed exists in users’ online social networks (OSNs) profiles for attractiveness-related self-esteem and need for popularity, and researchers further demonstrated that thin slices of OSN profiles, such as interest fields, can yield a stronger effect of unique impression management for global self-esteem. Relative to elders, young adults and teenagers tend to gain more popularity and better reach a comfortable level of recognition and connectedness by shaping their online identities (Dijck, 2015).

The hyperpersonal model (Walther, 1996) posits that in computer-mediated-communication (CMC) there are many opportunities for selective self-presentation and idealization. Due to reduced communication cues and potentially asynchronous communication in CMC, users may have more opportunities to control impressions of them. A few studies have explored impression management by status updates from SNSs. According to the quality of identity claims, individuals may make some statements about how they wish to be perceived. Status updates, as one type of identity claim, may thus also be associated with users’ desire to manage impressions. These frameworks offer us an approach to understanding impression management in posters’ status updates, based on the assumption that this kind of management goes on. Specifically, targets’ desired impression of the Big Five personality traits will be expected to significantly differ from their self-ratings, and their meta-perception, or awareness of how others view them, in terms of the Big Five may significantly correlate with their desired impression.

#### Meta-Perception: People’s Awareness of How Others View Them

People’s awareness of how others view them is meta-perception of themselves and the accuracy of these meta-awareness is then meta-accuracy (Laing et al., 1966; Kenny and Depaulo, 1993; Kenny, 1994). Researchers exploring meta-perception have demonstrated that people are generally knowledgeable about how others view them. On average, participants exhibited robust meta-accuracy for close acquaintances (Carlson and Furr, 2012), for instance, people who were well-adjusted interpersonally (e.g., socially skilled) had insight into what made them distinctive in their friends’ eyes (Carlson, 2016). Among strangers, people showed significant meta-accuracy for judgments on extraversion, agreeableness, and conscientiousness (Moritz and Roberts, 2017). However, in online contexts, the findings are mixed. Stopfer et al. (2014) found that targets knew how they were viewed by others for all traits except neuroticism based on online social network profiles; however, Facebook users believed that their profile postings made a more positive impression than they really did (Carlson, 2015). Essentially, however, meta-perception in an online context has not been studied adequately. Thus, the current study explores people’s meta-perception of their own Big Five personality traits based on the status updates they posted.

Notably, status updates as one type of identity claim are directed not only to others but also to posters themselves, as symbolic statements made by individuals sometimes for their own benefit, and they may also tend to reinforce their self-views (Gosling et al., 2002). Therefore, we hypothesize that status update posters have generally clear insight about what is conveyed to others; in other words, their meta-perception of their Big Five personality will be largely accurate.

#### Personality Judgment at Zero Acquaintance in an Online Context

Judgment accuracy refers to consistency between the impressions formed by observers based on several cues and the actual traits of targets. Consensus represents the agreement of independent observers on personality impressions according to a series of cues (Gosling et al., 2007). Both judgment accuracy and consensus can indicate that cues reflect the traits of the targets accurately.

Several studies have indicated that unfamiliar laypeople are able to accurately judge others’ personality traits based on subtle cues, such as a person’s natural stream-of-consciousness essays (Holleran and Mehl, 2008), textual information explicitly covering major life domains (Borkenau et al., 2016), photos of facial expressions (Sutherland et al., 2015; Walker and Vetter, 2016), video clips of someone introducing themselves (Hirschmüller et al., 2017), and other incidental cues such as photographs of someone’s shoes (Gillath et al., 2012), information on their music preferences (Rentfrow and Gosling, 2006), and images of their bedrooms and offices (Gosling et al., 2002).

A body of research has indicated that online information can reflect users’ personality traits accurately. Darbyshire et al. (2016) found that perceivers were able to accurately judge targets’ openness and conscientiousness based on their Facebook profiles. Further, personality traits can also be inferred from online social network profiles (Stopfer et al., 2014), selfies from Sina Weibo profile pictures (Qiu et al., 2015), identity claims on personal websites (Vazire and Gosling, 2004), linguistic cues from targets’ tweets (Qiu et al., 2012) and video clips on YouTube (Biel and Gatica–Perez, 2012). Taken together, these findings indicate that several different sources of information in an online context are valid cues for people to judge others’ personality even despite not being acquainted. Therefore, we believe that people’s status updates will also reflect their personality information.

Gosling et al.’s (2002) model emphasizes that identity claims are other-directed. The statements individuals made may convey truthful messages about what the individual is really like. Consistent with this, status updates as a new form of “one-to-many communication,” in which SNS users express what is on their mind (Deters and Mehl, 2013) may also reflect accurate Big Five personality traits information. Further, according to the lens model (Brunswik, 1956), perceivers use several perceivable cues in a given situation to infer the personality traits of targets. Three elements are vital to this judgment process: cue validity, which is the extent of association between the given cues and personality traits; cue utilization, which indicates the degree to which perceivers using the given cues to judge a personality trait; and sensitivity, which refers to perceivers’ ability to distinguish differences across cues. This model serves as a base for us to understand the association between status updates, personality traits, and interpersonal perception.

Accordingly, we propose that targets’ status updates can provide rich information to perceivers about their Big Five personality traits, and that perceivers are capable of making judgments about targets.

## Materials and Methods

### Participants

We recruited 131 young adults from the Southwest University of China as targets; 30 participants were male, and all participants were 18 to 25 years old (*M* = 20.65, *SD* = 1.70). Some participants were excluded as they had less than 20 status updates posted on WeChat and QQ; thus, 123 of these “target” participants were ultimately included.

In the personality judgment phase, we recruited 120 students from Southwest University of China as perceivers; 34 were male, and all were 18 to 25 years old. We recruited perceivers through online messages and provided them with a list of target names. They were asked whether they knew the targets before they came to the laboratory.

Thus, we had two groups of users: one regarded as targets, who needed to provide their own status updates as judgment materials, and one group of perceivers, whose mission was to evaluate the personality traits of targets based on those status updates.

This study was approved by the Ethics Committee of Southwest University of China. All 251 participants provided online consent both prior to participation and after debriefing and were informed that their participation was completely voluntary and that they could withdraw from the study any time.

### Measures

#### The Big Five

Targets rated their own personality using the Chinese version of the 44-item Big Five Personality Inventory (BFI-44; John et al., 1991), which measures extraversion, agreeableness, conscientiousness, neuroticism, and openness on a scale ranging from 1 (disagree strongly) to 5 (agree strongly). The Cronbach’s α in our sample for extraversion, agreeableness, conscientiousness, neuroticism, and openness were 0.85, 0.66, 0.79, 0.78, and 0.78, respectively.

The perceivers reported the personality traits of targets by completing a modified version of the BFI-44 after they observed the stimulus from targets (Stopfer et al., 2014).

#### Meta-Perception

Targets used another modified version of the BFI-44 to rate meta-perception. They were instructed as follows: “Please infer how other people will judge you when they come across your status updates.” Example items included “When other people come across my status updates, they will consider me to be someone who is energetic” (extraversion) and “when other people come across my status updates, they will consider me to be someone who gets nervous easily” (neuroticism).

#### Desired Impression of Personality Traits

Desired impression of Big Five personality traits was measured via another modified version of BFI-44. We asked targets to “imagine to how you wish to be judged when others come across your status updates.” Example items include “I wish others to consider me to be someone who is energetic when they come across my status updates” (extraversion) and “I wish others to consider me to be someone who gets nervous easily when they come across my status updates” (neuroticism).

### Procedure

The outline of the current study is as follows: one set of users, as targets, provided their status updates as judgment materials and submitted their self-report on the Big Five personality traits, and after 1 week, we collected their meta-perceptions and desired impressions via an online survey; another group of users, as perceivers, made personality judgments on the targets after observing their status updates.

#### Phase 1: materials and collection of self-reported personality data

Participants presented to the laboratory and took screenshots of their last 20 status updates from WeChat and QQ (most commonly used). These stimuli would be judged by perceivers in Phase 3. During this visit, we also collected their self-reports on the Big Five personality traits.

#### Phase 2: meta-perception data collection

To avoid confusion between self-view, meta-perception, and the targets’ desired impression of the Big Five personality traits, we did not collect the meta-perception or desired impression of Big Five personality traits in Phase 1; instead, after 1 week, we invited the participants from phase 1 to complete an online survey of meta-perception and desired impression via the popular Chinese professional survey website Wenjuanxing (^1^a website similar to SurveyMonkey). Because some targets at Phase 1 were unwilling to provide data for the second time, we only collected 120 participants’ meta-perception and desired impression of their Big Five personality traits.

#### Phase 3: personality judgments

Before Phase 3, we edited the screenshots of the target participants to only include the main contents of the status updates and removed other information such as avatars, users’ names, time of status updates, likes and comments, and other interaction with friends. We attempted to maintain naturalistic quality of materials to provide perceivers with more natural situations. There were 123 targets, each of whom provided 20 status updates from WeChat and QQ; therefore, in total, we had 2460 status updates. We then randomly divided the 123 targets into eight groups, with each group containing 14–16 targets, and it suggested that each group contained 280–300 status updates. Next, we randomly divided perceivers into eight groups with 14–16 perceivers in each group. We randomly matched one group of targets with one group of perceivers; thus, each target was judged by 14–16 perceivers.

Perceivers arrived at the laboratory and sat in front of a computer. They were instructed to observe all 20 status updates for one target before making judgments. There were no time restrictions for making the judgment.

## Results

### The Desire to Make a Specific Impression in One’s Status Updates

Since not all variables are normally distributed, the non-parametric tests are adopted in this study. To assess whether targets aimed to make a good impression in their status updates, we first performed a Wilcoxon signed-rank test and compared targets’ desired impression and self-view of personality traits (Table 1). The results showed that targets’ desired conscientiousness, neuroticism, and openness were significantly higher than their self-view of these traits. Their agreeableness was significantly lower than their desired impression, whereas desired impression of extraversion did not significantly differ from their self-view. In addition, if the targets did attempt impression management, it seemed that they might subjectively think that others perceive them as they wished to be perceived. Therefore, we calculated the correlations between their meta-perceptions and desired impressions. The results showed that their meta-perceptions were significantly correlated with their desired impressions for conscientiousness (*r* = 0.35, *p* < 0.001) and openness (*r* = 0.74, *p* < 0.001), whereas their meta-perception of extraversion (*r* = 0.16, *p* = 0.09), agreeableness (*r* = 0.11, *p* = 0.25), and neuroticism (*r* = 0.05, *p* = 0.59) did not significantly correlate with their desired impressions.

**Table 1:** Wilcoxon Signed-Rank Test between desired impression and self-ratings.

	Mean		Median		*Z*	Sig
	Desired impression	Self-view	Desired impression	Self-view		
Extraversion	25.94	26.77	26.00	27.00	-0.86	0.391
Agreeableness	29.86	33.90	25.51	34.00	-3.72	<0.000
Conscientiousness	36.62	29.94	37.00	29.00	-8.38	<0.000
Neuroticism	26.26	24.13	31.37	24.00	-2.14	0.032
Openness	39.42	36.53	39.00	37.00	-5.80	<0.000

### The Accuracy of Meta-Perception

Meta-accuracy was measured by correlating perceivers’ ratings and targets’ meta-perception. Back et al. (2010) stated that using aggregate perceiver ratings may overestimate the levels of agreement. Therefore, we not only calculated the correlations between the overall perceivers’ average ratings and target meta-perception, but also correlated each individual perceiver rating and targets’ meta-perception. The results for both the single and average perceiver ratings revealed significant correlations for extraversion, agreeableness, conscientiousness, and neuroticism, but not openness (Table 2).

**Table 2:** Single perceiver ratings and average perceiver ratings for all five traits.

	Meta-accuracy	
	Single perceiver ratings	Average perceiver ratings
Extraversion	0.17^∗∗∗^	0.29^∗∗^
Agreeableness	0.15^∗∗∗^	0.29^∗∗^
Conscientiousness	0.14^∗∗∗^	0.30^∗∗^
Neuroticism	0.13^∗∗∗^	0.28^∗∗^
Openness	0.06^∗^	0.10 (*p* = 0.26)

*^∗^p < 0.05; ^∗∗^p < 0.01; ^∗∗∗^p < 0.001.*

### Judgment Accuracy and Consensus

To assess judgment accuracy, self-other agreement was first calculated by correlating the self-reported scores on the BFI-44 and every single perceiver rating, and then we correlated the targets’ self-view scores with average ratings of each subset of perceivers (similar to Fong and Mar, 2015). The results (Table 3) both showed that there were significant correlations for extraversion, agreeableness, conscientiousness, neuroticism, and openness, reflecting a significant level of accuracy.

**Table 3:** Single perceiver ratings and average perceiver ratings for all five traits.

Judgment accuracy (self-other agreement)		
	**Single perceiver rating**	**Average perceiver ratings**
Extraversion	0.15^∗∗^	0.27^∗∗^
Agreeableness	0.15^∗∗^	0.23^∗^
Conscientiousness	0.07^∗∗^	0.17 (*p* = 0.06)
Neuroticism	0.15^∗∗^	0.27^∗∗^
Openness	0.09^∗∗^	0.20^∗^

*Judgment accuracy indicates the correlations between the self-ratings and observers’ average ratings. We also considered single perceiver ratings. ^∗^p < 0.05; ^∗∗^p < 0.01.*

In the second step, the consensus of each subset of observers was calculated via intra-class correlations (ICC, Vazire and Mehl, 2008), which reflected the consensus among different perceivers by testing; the consistency of judgment from different perceivers in one group. The results (Table 4) revealed that although each subset of targets and observers was distinct, all perceivers reached a significant consensus on all five personality traits. Users’ status updates postings on WeChat and QQ can reflect consistent personality information to different perceivers.

**Table 4:** Eight subsets of observers’ consensus for five traits.

	Intra-class correlations (ICC)								
		1	2	3	4	5	6	7	8
Extraversion	Average observer	0.89^∗∗∗^	0.81^∗∗∗^	0.79^∗∗∗^	0.73^∗∗∗^	0.95^∗∗∗^	0.84^∗∗∗^	0.82^∗∗∗^	0.88^∗∗∗^
	Single observer	0.33^∗∗∗^	0.22^∗∗∗^	0.21^∗∗∗^	0.16^∗∗∗^	0.55^∗∗∗^	0.26^∗∗∗^	0.24^∗∗∗^	0.33^∗∗∗^
Agreeableness	Average observer	0.90^∗∗∗^	0.79^∗∗∗^	0.86^∗∗∗^	0.26^∗^	0.91^∗∗∗^	0.89^∗∗∗^	0.91^∗∗∗^	0.82^∗∗∗^
	Single observer	0.36^∗∗∗^	0.19^∗∗∗^	0.30^∗∗∗^	0.03^∗^	0.40^∗∗∗^	0.35^∗∗∗^	0.40^∗∗∗^	0.25^∗∗∗^
Conscientiousness	Average observer	0.68^∗∗∗^	0.80^∗∗∗^	0.61^∗∗∗^	0.54^∗∗^	0.69^∗∗∗^	0.80^∗∗∗^	0.76^∗∗∗^	0.84^∗∗∗^
	Single observer	0.12^∗∗∗^	0.20^∗∗∗^	0.10^∗∗∗^	0.08^∗∗^	0.13^∗∗∗^	0.21^∗∗∗^	0.18^∗∗∗^	0.27^∗∗∗^
Neuroticism	Average observer	0.87^∗∗∗^	0.77^∗∗∗^	0.76^∗∗∗^	0.63^∗∗∗^	0.94^∗∗∗^	0.83^∗∗∗^	0.84^∗∗∗^	0.83^∗∗∗^
	Single observer	0.30^∗∗∗^	0.18^∗∗∗^	0.18^∗∗∗^	0.11^∗∗∗^	0.52^∗∗∗^	0.25^∗∗∗^	0.26^∗∗∗^	0.26^∗∗∗^
Openness	Average observer	0.82^∗∗∗^	0.43^∗^	0.64^∗∗∗^	0.56^∗∗^	0.80^∗∗∗^	0.85^∗∗∗^	0.37 (*p* = 0.08)	0.58^∗∗^
	Single observer	0.23^∗∗∗^	0.05^∗^	0.11^∗∗∗^	0.08^∗∗^	0.21^∗∗∗^	0.28^∗∗∗^	0.04 (*p* = 0.08)	0.09^∗∗^

*The intra-class correlations (ICC) refer to the consensus among different judges. The average observer means that the mean of all ratings is the unit of analysis; that is, average measure reliability gives the reliability of the mean of all raters’ ratings. The single observer means that individual ratings constitute the unit of analysis; that is, single measure reliability gives the reliability for a single judge’s rating. In addition, 1, 2, 3… indicate group 1, group 2, group 3… as the subsets of perceivers. ^∗^p < 0.05; ^∗∗^p < 0.01; ^∗∗∗^p < 0.001.*

### Unsuccessful Impression Management

Based on the results for meta-accuracy and judgment accuracy, we may conclude that although targets hope that they can make a different impression for most traits in their status updates and think that they can make a good impression for conscientiousness and openness, the actual results for the meta-accuracy and judgment accuracy show that their status updates reflect their actual personality. Users may attempt to make a different impression from their actual personality, but this attempt does not influence perceivers’ judgments.

To support this assumption, we compared the differences between desired impressions and perceiver’ judgments using a Mann–Whitney *U*-test. For all five traits, there were significant differences between targets’ desired impressions and observers’ judgments (Table 5). Additionally, to further examine the effects of impression management on observers’ ratings, we conducted a series of regression analyses to test the extent to which the desired impression contributed to the perceivers’ ratings (similar to Vazire and Gosling, 2004). The results indicated that after removing the self-view component from the desired self-ratings, there were no traits displaying significant evidence of impression management, except for extraversion, because there was no impression management for this trait (Table 6). To summarize, targets’ attempts to manage impressions were unsuccessful.

**Table 5:** Mann–Whitney Test between desired impression and perceivers-ratings.

		Mean rank			
	Mann– Whitney *U*	Desired impression	Perceiver ratings	*Z*	Sig
Extraversion	5672.50	107.77	133.23	-2.84	0.004
Agreeableness	6247.00	112.56	128.44	-1.77	0.076
Conscientiousness	1493.50	168.05	72.95	-10.62	<0.001
Neuroticism	5460.50	135.00	106.00	-3.24	0.001
Openness	2605.00	158.79	82.21	-8.55	<0.001

**Table 6:** Regression of perceivers’ ratings on desired impression and self-view: Standardized Beta Weights.

	Step 1	Step 2	
	Self-view	Self-view	Desired impression
Extraversion	0.13^**^	0.17^***^	-0.09^*^
Agreeableness	0.23^***^	0.23^***^	0.03
Conscientiousness	0.07 (*p* = 0.07)	0.05	0.06
Neuroticism	0.13^**^	0.13^**^	0.02
Openness	0.06	0.03	0.05

*The self-view was entered in the first block of the regression equation, and the ideal impression was entered in the second block. ^∗^p < 0.05; ^∗∗^p < 0.01; ^∗∗∗^p < 0.001.*

## Discussion

The current research investigated whether posters used impression management in their status updates, whether their impression management was successful, whether posters were aware of how perceivers viewed them based on their status updates, and whether observers were able to accurately judge targets’ personality traits according to the status updates.

### Understanding Users’ Impression Management

The current study extends the existing findings on impression management in an online context. The results of the Wilcoxon signed-rank test revealed that users wish to make an impression in their status updates that differs from how they actually see themselves, hoping that others would judge them as more conscientious, neurotic, and open, but less agreeable. Moreover, the significant correlations between targets’ meta-perception and desired impression for conscientiousness and openness also indicate that they might subjectively think that they can make a positive impression on others for these two traits; these findings are in line with previous studies suggesting that people wish to be judged positively (e.g., Manago et al., 2008; Dijck, 2015). Before users post their status updates, they have plenty of time to edit and revise the message in the post and choose photographs to convey what they want. There is little time restriction; thus, people may have many opportunities to control their impression in an online context.

Our results support the hyperpersonal model, which proposes that CMC users selectively self-present, revealing information about themselves in a selective, controlled way that treats information as malleable to achieve socially desirable effect (Walther, 1996). Further, our findings also support Gosling et al.’s (2002) model which stated that individuals indeed try to make some statements online about how they wish to be regarded. Users in the current study wish to make a different impression on others via their status updates.

However, surprisingly, targets did not wish to make a positive impression for agreeableness, and neuroticism. They even hoped that others would think of them as more neurotic and less agreeable. We suggest that this might be due to the privacy settings on SNSs, by which they can choose which friends can see their status updates. As times, they might use negative words to express their bad mood or other negative information. They might consider SNSs to be platforms to express their negative feelings and therefore might not desire to make a good impression for all traits. Future research should conduct in-depth interviews on users’ real thoughts about what they wish to express in status updates.

### Understanding Meta-Accuracy, Judgment Accuracy, and Consensus

The findings related to meta-accuracy reveal that of the Big Five, only openness was not accurately inferred by targets at average-perceiver level. People hope they can make a different impression on others; however, their meta-accuracy was high. Individuals hope to make a good or bad impression on others, but they know what they are actually expressing in their status updates, therefore, they can accurately infer how others judge them when others come across their status updates. This consistent with Gosling et al.’s (2002) description of the model of interpersonal perception, status updates make an identity claims not only on others but also on posters themselves; these symbolic statements made by individuals may sometimes be for their own benefit, and reinforce their self-views.

The findings on judgment accuracy reveal that perceivers can accurately judge targets’ extraversion, agreeableness, conscientiousness, neuroticism, and openness. This is partially in line with, but broader than prior findings based on tweets (Twitter messages), which indicated that observers were able to accurately judge owners’ agreeableness and neuroticism (Qiu et al., 2012). The differences between these results likely suggest that different contents expressed in an online context may provide different information related to personality. The status updates in our study contain not only original written messages by the target participants but also photographs and reposted materials such as music or articles different from studies that only use linguistic cues, such as from tweets (Qiu et al., 2012) or images, such as selfies from microblog profile pictures (Qiu et al., 2015). The results on judgment consensus, however, are in line with findings from previous research examining tweets (Qiu et al., 2012) and selfies (Qiu et al., 2015): all Big Five personality traits, including extraversion, agreeableness, conscientiousness, neuroticism, and openness, can be judged consistently by different subsets of non-acquainted perceivers. This indicates that the content people choose to post can give rise to a consistent impression among different perceivers. In this sense, the current findings again support Gosling et al.’s (2002) model: users’ status updates indeed reflect their truthful personality information. Further, our results also support the lens model (Brunswik, 1956), because our results suggested that status updates on SNSs are valid cues, and perceivers are capable enough of making judgments based on targets’ status updates.

### Unsuccessful Impression Management

Our results demonstrate that users’ impression management via status updates is basically unsuccessful, as it does not influence perceivers’ judgment. If so, why? There are several possible explanations. The first involves the nature of the SNSs. Unlike some microblog platforms such as Sina Weibo in which strangers can browse users’ posts without restriction, the status updates posted on WeChat and QQ are presented to people who knows the poster, and users and their online friends may also interact with each other offline. Therefore, users must maintain a consistent impression formed by their friends in both online and offline contexts; thus, they might not dare to manipulate their impression too actively, leaving more room for true information alongside managed information. This is in line with Vazire and Gosling (2004), who suggest that people manage their online expressions within a certain parameter of their offline personality. Walther and Parks (2002) similarly noted that due to the less anonymous quality of these platforms, radical departures of online and physical self are less possible.

In the case of perceivers, they may not completely trust the content of status updates. Krämer et al. (2017) noted that observers become suspicious when they perceive “presenters” as motivated to manage impression of their selfies, and according to the lens model of Brunswik (1956), perceivers may take motives of the users into account in order to improve accuracy of perception.

The warranting theory (Stone, 1995; Walther and Parks, 2002) posits that a warrant is online information that creates a connection between the online and offline self, and that the online and offline selves represent a continuum rather than mutually exclusive selves. Walther and Parks (2002) defined the warranting value of information about a person based on the perceiver’s perception about the extent to which the content of that information is immune to manipulation by the sender. This means that perceivers tend to trust information which is difficult for senders to manipulate. Perceivers often make efforts to seek information in order to connect senders’ online and offline selves — not blindly making judgments based on status updates but also making efforts to seek information that can link targets’ offline and online selves. When making judgments, perceivers may deeply consider how the target behaves in the offline world and may base their information off more than is shown overtly.

Taken together, the present findings make important theoretical and practical contributions. Regarding theoretical significance, our study is the first to explore users’ meta-perception and perceivers’ perception of strangers’ Big Five personality dimensions based on status updates, and the accuracy of judgment offers new empirical evidence on personality expression in SNSs. Our study expanded the number of observers from less than 10 in most previous studies (Gosling et al., 2007; Qiu et al., 2012; Wall et al., 2016) and the consensus of observers reached a significant level for each Big Five trait in each of our subsets. These results strengthened previous studies’ findings that a consistent impression of someone’s personality can be formed among different, separate perceivers. In this study, we novelly considered users’ meta-perception and perceivers’ judgment accuracy to illustrate the process of impression management of status updates, and gathered new evidence supporting the hyperpersonal model and the model about interpersonal perception. That impression management was unsuccessful also supports Brunswik’s lens model and warranting theory, which supposes that perceivers use cues to make judgments in a more sophisticated way, rather than simply using surface information. The current study also has some practical implications. With the increasing prevalence of SNSs and the increasing frequency of status update postings, understanding the processes of impression management by users and impression formation by perceivers can help people to understand their online friends and promote relationship development.

### Limitations and Future Directions

There are some limitations to the current study. The first one is that as the participants were mainly university students, their status updates might express a relatively narrow range of topics and information compared to people in general. Hence, future studies should extend the age range of samples to further explore perceivers’ judgment accuracy based on status updates. At the same time, we did not report on gender differences in our research, whereas prior research has shown that males and females choose different profile pictures on Facebook for presenting themselves (Zheng et al., 2016). Females are more likely to use their profile pictures for impression management than are males, and they also tend to spend more time than do males on photo activities (McAndrew and Jeong, 2012). Given these findings, we believe other gender differences may also exist in this area; future studies should consider potential differences in depth with respect to what males and females post in status updates, meta-accuracy, and impression management. Second, our stimulus materials excluded likes, comments, and interactions between users and their friends. This content may reflect more authentic information about people’s personality, as according to the warranting principle other-generated information, such as comments on posts, has more effect on perceivers’ judgments than target self-descriptions do (Walther et al., 2009). Impression management may thus also occur in comments and interactions between users and their friends. Future research can more deeply investigate these possibilities. Third, in the current study, we focused on the Big Five personality traits, but there are many other dimensions worthy of exploration, such as meta-perception and perceivers’ judgments of self-esteem, loneliness, and humility, and whether these perceptions differ between online and offline contexts. Our findings demonstrate phenomena regarding unsuccessful impression management in perceivers’ perceptions, and future studies should investigate the potential mechanisms of unsuccessful impression formation through status updates or other information in an online context. Finally, future studies can consider users’ meta-perceptions and perceivers’ perceptions to explore the inherent mechanisms of impression management and impression formation on a deeper level. In addition, attention should also be paid to qualitative research, although the current study showed that impression management occurs in users’ status updates, the content of status updates can reflect people’s actual personality traits, and therefore, future studies should investigate which cues are important for perceivers’ judgments and which cues actually reflect users’ personality traits.

## Conclusion

The current study made use of information derived from status updates by SNS users. When users post status updates in SNSs, they tend to actively manage their personality impression even if the updates will be presented only to friends who already know them well. Although users may hope to make a certain impression on others, they tend to know what they are expressing in their status updates and that it might not match that impression. That is, they can accurately infer how others view them based on their status updates. Unexpectedly, impression management does not influence perceivers’ judgment process or results; these observers are able to accurately judge targets’ Big Five traits based on their status updates. Perhaps users are aware that their posts will be observed by their friends, and therefore ensure that their impression management is within certain parameters of their offline self; or perhaps perceivers recognize the user’s motivation of impression management, and therefore suspect the authenticity of the status updates and make more sophisticated and careful judgments.

## Ethics Statement

This study was approved by the Ethics Committee of Southwest University of China. All participants were informed that their participation was completely voluntary and that they may withdraw from the study any time.

## Author Contributions

TW proposed the research idea, designed and carried out the present study, as well as drafted the manuscript. YZ participated in the design and experimental work and interpretation, and revised this article critically.

## Conflict of Interest Statement

The authors declare that the research was conducted in the absence of any commercial or financial relationships that could be construed as a potential conflict of interest.

## Footnotes

1 www.sojump.com

## References

[B1] Back M. D. Stopfer J. M. Vazire S. Gaddis S. Schmukle S. C. Egloff B. (2010). Facebook profiles reflect actual personality, not self–idealization. *Psychol. Sci.* 21 372–374. 10.1016/j.jrp.2018.08.007 20424071

[B2] Biel J. I. Gatica–Perez D. (2012). The youtube lens: crowdsourced personality impressions and audiovisual analysis of vlogs. *IEEE Trans. Multimedia* 15 41–55. 10.1109/tmm.2012.2225032

[B3] Borkenau P. Mosch A. Tandler N. Wolf A. (2016). Accuracy of judgments of personality based on textual information on major life domains. *J. Pers.* 84 214–224. 10.1111/jopy.12153 25417640

[B4] Brunswik E. (1956). *Perception and the Representative Design of Psychological Experiments.* Berkeley, CA: University of California Press.

[B5] Carlson C. L. (2015). How do facebook users believe they come across in their profiles?: a meta–perception approach to investigating facebook self–presentation. *Commun. Res. Rep.* 32 93–101.

[B6] Carlson E. N. (2016). Do psychologically adjusted individuals know what other people really think about them? The link between psychological adjustment and meta–accuracy. *Soc. Psychol. Pers. Sci.* 7 717–725. 10.1177/1948550616646424

[B7] Carlson E. N. Furr R. M. (2012). Resolution of meta–accuracy should people trust their beliefs about how others see them? *Soc. Psychol. Pers. Sci.* 4 419–426. 10.1177/1948550612461653

[B8] Darbyshire D. Kirk C. Wall H. J. Kaye L. K. (2016). Don’t judge a (face)book by its cover: exploring judgement accuracy of others’ personality on facebook. *Comput. Hum. Behav.* 58 380–387. 10.1016/j.chb.2016.01.021

[B9] Deters F. G. Mehl M. R. (2013). Does posting facebook status updates increase or decrease loneliness? An online social networking experiment. *Soc. Psychol. Pers. Sci.* 4 579–586. 10.1177/1948550612469233 24224070 PMC3820167

[B10] Dijck J. V. (2015). ‘You have one identity’: performing the self on facebook and linkedin. *Libri* 35 199–215. 10.1177/0163443712468605

[B11] Fong K. Mar R. A. (2015). What does my avatar say about me? Inferring personality from avatars. *Pers. Soc. Psychol. Bull.* 41 237–249. 10.1177/0146167214562761 25576173

[B12] Gillath O. Bahns A. J. Ge F. Crandall C. S. (2012). Shoes as a source of first impressions. *J. Res. Pers.* 46 423–430. 10.1016/j.jrp.2012.04.003

[B13] Goffman E. (1959). *The Presentation of Self in Everyday Life.* Newyork, NY: Doubleday/Anchor Books.

[B14] Gosling S. D. Gaddis S. Vazire S. (2007). Personality impressions based on facebook profiles in *Paper presented at 2007 International Conference on Weblogs and Social Media, ICWSM 2007*, Boulder, CO.

[B15] Gosling S. D. Ko S. J. Mannarelli T. Morris M. E. (2002). A room with a cue: personality judgments based on offices and bedrooms. *J. Pers. Soc. Psychol.* 82 379–398. 10.1037/0022-3514.82.3.379 11902623

[B16] Hirschmüller S. Schmukle S. C. Krause S. Back M. D. Egloff B. (2017). Accuracy of self–esteem judgments at zero acquaintance. *J. Pers.* 86 308–319. 10.1111/jopy.12316 28317118

[B17] Holleran S. E. Mehl M. R. (2008). Let me read your mind: personality judgments based on a person’s natural stream of thought. *J. Res. Pers.* 42 747–754. 10.1016/j.jrp.2007.07.011

[B18] John O. P. Donahue E. M. Kentle R. L. (1991). *The Big–Five Inventory.* Berkeley, CA: University of California.

[B19] Kenny D. A. (1994). *Interpersonal Perception: A Social Relations Analysis.* New York, NY: Guilford Press.

[B20] Kenny D. A. Depaulo B. M. (1993). Do people know how others view them? An empirical and theoretical account. *Psychol. Bull.* 114 145–161. 8346325 10.1037/0033-2909.114.1.145

[B21] Kowalski R. M. Leary M. R. (1990). Strategic self–presentation and the avoidance of aversive events: antecedents and consequences of self–enhancement and self–depreciation. *J. Exp. Soc. Psychol.* 26 322–336.

[B22] Krämer N. C. Feurstein M. Kluck J. P. Meier Y. Rother M. Winter S. (2017). Beware of selfies: the impact of photo type on impression formation based on social networking profiles. *Front. Psychol.* 8:188. 10.3389/fpsyg.2017.00188 28261129 PMC5311061

[B23] Krämer N. C. Winter S. (2008). Impression management 2.0: the relationship of self–esteem, extraversion, self–efficacy, and self–presentation within social networking sites. *J. Media Psychol.* 20 106–116.

[B24] Laing R. D. Phillipson H. Lee A. R. (1966). *Interpersonal Perception: A Theory and a Method of Research.* Oxford: Springer.

[B25] Lee E. Ahn J. Kim Y. J. (2014). Personality traits and self–presentation at facebook. *Pers. Individ. Differ.* 69 162–167.

[B26] Makhanova A. Mcnulty J. K. Maner J. K. (2017). Relative physical position as an impression–management strategy: sex differences in its use and implications. *Psychol. Sci.* 28 567–577. 10.1177/0956797616688885 28485703

[B27] Manago A. M. Graham M. B. Greenfield P. M. Salimkhan G. (2008). Self–presentation and gender on myspace. *J. Appl. Dev. Psychol.* 29 446–458. 10.1016/j.appdev.2008.07.001

[B28] Marshall T. C. Lefringhausen K. Ferenczi N. (2015). The big five, self–esteem, and narcissism as predictors of the topics people write about in facebook status updates. *Pers. Individ. Differ.* 85 35–40. 10.1016/j.paid.2015.04.039

[B29] McAndrew F. T. Jeong H. S. (2012). Who does what on Facebook? Age, sex, and relationship status as predictors of Facebook use. *Comput. Hum. Behav.* 28 2359–2365. 10.1016/j.chb.2012.07.007

[B30] Moritz D. Roberts J. E. (2017). Self–other agreement and metaperception accuracy across the big five: examining the roles of depression and self–esteem. *J. Pers.* 86 296–307. 10.1111/jopy.12313 28258585

[B31] Pounders K. Kowalczyk C. M. Stowers K. (2016). Insight into the motivation of selfie postings: impression management and self–esteem. *Eur. J. Mark.* 50 1879–1892.

[B32] Qiu L. Lin H. Ramsay J. Yang F. (2012). You are what you tweet: personality expression and perception on twitter. *J. Res. Pers.* 46 710–718. 10.1016/j.jrp.2012.08.008

[B33] Qiu L. Lu J. Yang S. Qu W. Zhu T. (2015). What does your selfie say about you? *Comput. Hum. Behav.* 52 443–449. 10.1016/j.chb.2015.06.032

[B34] Rentfrow P. J. Gosling S. D. (2006). Message in a ballad: the role of music preferences in interpersonal perception. *Psychol. Sci.* 17 236–242. 16507064 10.1111/j.1467-9280.2006.01691.x

[B35] Stone A. R. (1995). *The War of Desire and Technology at the End of the Mechanical Age.* Cambridge, MA: MIT Press.

[B36] Stopfer J. M. Egloff B. Nestler S. Back M. D. (2014). Personality expression and impression formation in online social networks: an integrative approach to understanding the processes of accuracy, impression management and meta–accuracy. *Eur. J. Pers.* 28 73–94. 10.1002/per.1935

[B37] Sutherland C. A. M. Rowley L. E. Amoaku U. T. Daguzan E. Kiddrossiter K. A. Maceviciute U. (2015). Personality judgments from everyday images of faces. *Front. Psychol.* 6:1616. 10.3389/fpsyg.2015.01616 26579008 PMC4621398

[B38] Tencent. (2017). *Tencent Releases its Fourth Quarter and Full–Year Results for 2017*. Available at: https://www.tencent.com/zh-cn/articles/8003481521633431.pdf (accessed May, 2018).

[B39] Tifferet S. Vilnai–Yavetz I. (2018). Self–presentation in linkedin portraits: common features, gender, and occupational differences. *Comput. Hum. Behav.* 80 33–48. 10.1016/j.chb.2017.10.013

[B40] Vazire S. Gosling S. D. (2004). e–Perceptions: personality impressions based on personal websites. *J. Pers. Soc. Psychol.* 87 123–132. 10.1037/0022-3514.87.1.123 15250797

[B41] Vazire S. Mehl M. R. (2008). Knowing me, knowing you: the accuracy and unique predictive validity of self–ratings and other–ratings of daily behavior. *J. Pers. Soc. Psychol.* 95 1202–1216. 10.1037/a0013314 18954202

[B42] Walker M. Vetter T. (2016). Changing the personality of a face: perceived big two and big five personality factors modeled in real photographs. *J. Pers. Soc. Psychol.* 110 609–624. 10.1037/pspp0000064 26348599

[B43] Wall H. J. Kaye L. K. Malone S. A. (2016). An exploration of psychological factors on emoticon usage and implications for judgement accuracy. *Comput. Hum. Behav.* 62 70–78. 10.1016/j.chb.2016.03.040

[B44] Walther J. B. (1996). Computer–mediated communication impersonal, interpersonal, and hyperpersonal interaction. *Commun. Res.* 23 3–43. 10.1177/009365096023001001

[B45] Walther J. B. Heide B. V. D. Hamel L. M. Shulman H. C. (2009). Self–generated versus other–generated statements and impressions in computer–mediated communication: a test of warranting theory using facebook. *Commun. Res.* 36 229–253. 10.1177/0093650208330251

[B46] Walther J. B. Parks M. R. (2002). “Cues filtered out, cues filtered in: computer–mediated communication and relationships,”in *Handbook of Interpersonal Communication* ed. Miller G. R. (Thousand Oaks, CA: Sage) 529–563.

[B47] Winter S. Neubaum G. Eimler S. C. Gordon V. Theil J. Herrmann J. (2014). Another brick in the facebook wall–How personality traits relate to the content of status updates. *Comput. Hum. Behav.* 34 194–202. 10.1016/j.chb.2014.01.048

[B48] Zheng W. Yuan C. H. Chang W. H. Wu H. C. Jim Y. C. (2016). Profile pictures on social media: gender and regional differences. *Comput. Hum. Behav.* 63 891–898. 10.1016/j.chb.2016.06.041

